# Non-alcoholic fatty liver disease is associated with hyperandrogenism in women with polycystic ovary syndrome

**DOI:** 10.1038/s41598-023-39428-4

**Published:** 2023-08-17

**Authors:** So-hyeon Hong, Yeon-Ah Sung, Young Sun Hong, Do Kyeong Song, Hyein Jung, Kyungah Jeong, Hyewon Chung, Hyejin Lee

**Affiliations:** 1 Division of Endocrinology and Metabolism, Department of Internal Medicine, Ewha Womans University College of Medicine, Seoul, South Korea; 2 Department of Obstetrics and Gynecology, Ewha Womans University College of Medicine, Seoul, South Korea; 3 Ewha Womans University Medical Center, 1071, Anyangcheon-ro, Yangcheon-gu, Seoul, South Korea

**Keywords:** Medical research, Risk factors

## Abstract

Polycystic ovary syndrome (PCOS) is a highly complex reproductive metabolic disorder and women with PCOS have high prevalence of non-alcoholic fatty liver disease (NAFLD). Despite both hyperandrogenism and insulin resistance are common pathophysiologies in NAFLD and PCOS, this association is still controversial. Therefore, the aim of this study is to evaluate the relationship between hyperandrogenism and NAFLD in females diagnosed with PCOS. We recruited 667 women diagnosed with PCOS and 289 women with regular menstrual cycles as control. The PCOS diagnosis was made using National Institute of Child Health and Human Disease criteria. Total and free testosterone levels (TT and TF, respectively), and free androgen index (FAI) were used as measures of hyperandrogenism. Fatty liver index and liver fat score (FLI and LFS, respectively), and hepatic steatosis index (HSI) were used to assess NAFLD. The prevalence of NAFLD in PCOS women evaluated by LFS, FLI, and HIS were 19.9, 10.3, and 32.2%, respectively. In the control group, the incidence was 2.1, 0.7, and 4.2%, respectively. Both FT and FAI levels showed significant association with increased NAFLD-related indices, after adjusting for insulin resistance and other factors (LFS (OR 3.18 (95% CI 1.53–6.63) in FT; 1.12 (1.04–1.22) in FAI), FLI (OR 2.68 (95% CI 1.43–5.03) in FT; 1.13 (1.06–1.20) in FAI), and HSI (OR 3.29 (95% CI 2.08–5.21) in FT; 1.5 (1.09–1.21) in FAI). TT did not exhibit association with any NAFLD index. In women with PCOS, significantly higher rate of NAFLD was observed compared to the control women. The FT and FAI were independently associated with NAFLD in women with PCOS. The findings suggest the possibility of hyperandrogenism contributing to the progression and/or development of NAFLD in PCOS.

## Introduction

Polycystic ovary syndrome (PCOS), a common endocrine disorder in reproductive-aged women, is characterized by oligomenorrhea, hyperandrogenism, and polycystic ovary morphology^1^. Insulin resistance is the main pathophysiology of PCOS and leads to an increased risk of diverse cardiometabolic diseases, including diabetes mellitus, dyslipidemia, cardiovascular disease, and non-alcoholic fatty liver disease (NAFLD)^2^.

NAFLD has recently emerged as the most common chronic liver disease worldwide^3^. An increasing number of reports suggest that women with PCOS have a higher risk of the development and progression of NAFLD compared to women without PCOS^4,5^. Like PCOS, insulin resistance is the main pathophysiology of NAFLD. Insulin resistance exacerbates hepatic *de-novo* lipogenesis, making the liver susceptible to injury by oxidative stress, which leads to necroinflammation, fibrosis, and the development of NAFLD^6^.

The association between hyperandrogenism and NAFLD in PCOS is complex and not clearly understood. Some studies have found that PCOS women with hyperandrogenism had higher hepatic steatosis compared to PCOS women with normal androgens, independent of insulin resistance^7,8^. However, other studies have reported that hyperandrogenism in PCOS women was not an independent risk factor for NAFLD, but rather mediated through insulin resistance^9,10^.

In addition, while the gold standard diagnosis of NAFLD is liver biopsy or MRI, it is costly and hard to perform in large population. Recently, simple and convenient markers, such as liver fat score (LFS), fatty liver index (FLI), and hepatic steatosis index (HSI) have been demonstrated to exhibit comparable coefficient values to standard NAFLD diagnosis^11–13^. However, studies utilizing these NAFLD indices in the PCOS population, particulary in young Asian groups are scarce. The use of these NAFLD indices in PCOS women may provide a useful tool to efficiently screen for NAFLD in a clinical setting.

In this study, we aimed to explore the relationship between hyperandrogenism assessed by total testosterone, calculated free testosterone, and free androgen index (FAI) and NAFLD indices assessed by liver fat score (LFS), fatty liver index (FLI), and hepatic steatosis index (HSI) and to examine whether the association is independent of insulin resistance or not in women with PCOS.

## Methods

### Participants

The study population consisted of 667 women with PCOS attending the endocrinology and gynecology clinic at Ewha Womans University Mokdong Hospital from December 2008 through December 2010. —The presence of hyperandrogenism and oligo- or amenorrhea^14^, as defined by the National Institute of Child Health and Human Disease (NICHD) criteria, was used to diagnose PCOS. Hyperandrogenism was defined as biochemical hyperandrogenemia, that is, a total testosterone level above the 95th percentile (total testosterone ≥ 2.3 nmol/L or free testosterone ≥ 0.029 nmol/L) in 1120 healthy regular cycling women^15,16^. Patients with other androgen-excess disorders, including congenital adrenal hyperplasia, Cushing’s syndrome, or androgen-secreting neoplasia, were excluded. A total of 289 healthy, regular cycling women with no family history of PCOS were recruited as controls. Control group also underwent blood tests to confirm the absence of hyperandrogenism, same as the PCOS group. Subjects treated with oral contraceptives or metformin for 3 months prior to enrollment were excluded. The detailed study protocol was described in a previous study^17^. Written informed consent was obtained from all participants and the Institutional Review Board of Ewha Women’s University Mokdong Hospital approved this study (IRB No. 187–30).

### Anthropometric and biochemical evaluation

All subjects were instructed to visit the clinic on the third day of their menstruation period following an overnight fast of at least 8 h. The height and weight of each subject were measured on a weighing scale (JENIX, Seoul, Korea) while standing barefoot in a straight position with light clothing.

We collected fasting venous blood samples and performed the 75 g oral glucose tolerance test. Plasma glucose levels were measured using the glucose oxidase method (Beckman Model Glucose Analyzer 2, CA, USA), and serum insulin levels were measured using a radioimmunoassay kit (Biosource, Nivelles, Belgium). Serum aspartate transaminase (AST), alanine aminotransferase (ALT), gamma-glutamyl transferase (GGT), and triglycerides (TG) were measured using an enzymatic assay performed with an automated analyzer (Hitachi 7150 Automatic Chemistry Analyzer, Tokyo, Japan). Insulin resistance was estimated using the homeostatic model assessment for insulin resistance (HOMA-IR) according to the following formula: fasting plasma glucose (mg/dL) × fasting serum insulin (IU/mL)/405^18^.

Total testosterone levels were measured using a commercial chemiluminescent immunoassay kit (Siemens, NY, USA), and sex hormone-binding globulin (SHBG) levels were measured by an immunoradiometric assay using a commercial kit (DPC, Los Angeles, CA, USA). Free testosterone levels were calculated from total testosterone, SHBG, and albumin, using the formula from the International Society for the Study of the Aging Male website^15^. FAI was calculated as total testosterone (nmol/L)/SHBG (nmol/L) × 100^19^.

We selected three NAFLD indices—LFS, FLI, and HSI—based on their frequent usage and research, as well as their development or validation specifically for the East Asian population. The LFS was calculated using the following formula: − 2.89 + 1.18 × metabolic syndrome (yes = 1/no = 0) + 0.45 × T2DM (yes = 2/no = 0) + 0.15 × fasting serum insulin (uU/mL) + 0.04 × AST (U/L) − 0.94 × AST/ALT and LFS >  − 0.640, was considered as having NAFLD^11^. In 470 Finnish individuals, the receiver operating curve of LFS was 0.87 compared to liver fat content using proton magnetic resonance spectroscopy. The optimal cut-off point of − 0.640 predicted a sensitivity of 86% and specificity of 71%^11^. The FLI was calculated using the following formula: 1/(1 + exp(− x)) × 100, x = 0.953 × loge (TG) + 0.139 × BMI + 0.718 × loge (GGT) + 0.053 × (waist circumference) − 15.745 and FLI > 60, was considered as having NAFLD^12^. It was developed in 596 Italin subjects and the receiver operating curve of FLI was 0.85 compared to NAFLD diagnosed on ultrasonography^12^. The HSI was calculated using the following formula: 8 × AST/ALT + BMI (+ 2 if diabetes mellitus, + 2 if female) and HSI > 36, was considered as having NAFLD^13^. HSI was derived in Korean subjects and the receiver operating curve of HSI was 0.812^13^. Those indices were performed validation in the other study groups and verified the diagnostic accuracy of NAFLD^20–23^.

### Statistical analysis

Quantitative variables are presented as the mean ± standard deviation, and comparisons of two groups were performed using the Student’s unpaired *t*-test. Categorical variables were analyzed by the χ^2^ test.

We calculated the BMI-adjusted *P-*value as an additional analysis because BMI has an impact on NAFLD, metabolic parameters, and testosterone. A multiple logistic regression analysis was performed to assess the variables that were predictive for NAFLD in women with PCOS. In considering the adjusting factors, we conducted univariate analysis to examine the association between NAFLD, hyperandrogenism, and multiple confounding factors, including age, BMI, AST, ALT, GGT, fasting glucose, HOMA-IR, and triglyceride. All of these confounding factors were statistically associated with NAFLD. However, since each NAFLD index was calculated using different variables, there is a potential statistical issue of double adjustment if all variables are included as covariates. Therefore, for the LFS index, we adjusted for age, BMI, and HOMA-IR, excluding AST, ALT, TG, and fasting glucose that were used in the calculation equation. For the FLI, we adjusted for age, AST, ALT, fasting glucose, and HOMA-IR, excluding BMI and TG used in the calculation equation. Lastly, for the HSI index, we adjusted for age, TG, fasting glucose, and HOMA-IR, excluding BMI, AST, and ALT used in the calculation equation. *P* < 0.05 was considered to be statistically significant for all analyses. The statistical analysis was performed using the SPSS 20.0 software package (IBM Corp., Chicago, IL, USA).

### Ethics approval and consent to participate

This study was approved by the Institutional Review Board of Ewha Women’s University Mokdong Hospital (IRB No. 187–30). We obtained written informed consent from all participants, and conducted according to the Helsinki Declaration.

## Results

### Baseline characteristics

The clinical and biochemical characteristics of women with PCOS and controls are presented in Table 1. In women with PCOS, the prevalence of NAFLD was 19.9% in LFS, 10.3% in FLI, and 32.2% in HSI compared to control − 2.1% in LFS, 0.7% in FLI, and 4.2% in HSI. The women with PCOS also had higher BMI, systolic and diastolic blood pressure, AST, ALT, GGT, TG, fasting glucose, insulin, and HOMA-IR levels. Adjusting for BMI, differences in diastolic blood pressure, AST, and fasting glucose levels were not significant.

**Table 1:** Basal characteristics of study subjects according to PCOS.

	PCOS (n = 667)	Control (n = 289)	*P* value (unadjusted)	*P* value (BMI-adjusted)
NAFLD-related index				
LFS	− 1.28 ± 1.77	− 2.75 ± 0.94	< 0.01	< 0.01
FLI	19.1 ± 24.8	5.6 ± 7.7	< 0.01	< 0.01
HSI	33.8 ± 6.3	28.8 ± 3.3	< 0.01	< 0.01
NAFLD (n (%))				
LFS	133 (19.9)	6 (2.1)	< 0.01	< 0.01
FLI	69 (10.3)	2 (0.7)	< 0.01	0.33
HSI	215 (32.2)	12 (4.2)	< 0.01	< 0.01
Age (years)	25 ± 5	25 ± 5	0.9	0.37
BMI (kg/m^2^)	23.5 ± 4.7	21.1 ± 2.7	< 0.01	
WC (cm)	78.6 ± 11.4	72.8 ± 7.2	< 0.01	0.29
SBP (mmHg)	111 ± 12	107 ± 9	< 0.01	0.03
DBP (mmHg)	71 ± 10	69 ± 8	< 0.01	0.62
AST (U/L)	23 ± 15	21 ± 7	< 0.01	0.93
ALT (U/L)	24 ± 24	15 ± 10	< 0.01	< 0.01
GGT (U/L)	16 ± 12	11 ± 6	< 0.01	< 0.01
TG (mg/dL)	98 ± 58	74 ± 33	< 0.01	< 0.01
Fasting glucose (mg/dL)	87 ± 14	85 ± 7	< 0.01	0.19
Fasting insulin (uU/mL)	10.7 ± 7.4	4.7 ± 4.8	< 0.01	< 0.01
HOMA-IR	2.37 ± 2.07	1.01 ± 1.05	< 0.01	< 0.01
Total testosterone (ng/dL)	77.4 ± 18.0	42.7 ± 12.1	< 0.01	< 0.01
Free testosterone (ng/dL)	1.12 ± 0.43	0.38 ± 0.16	< 0.01	< 0.01
Free androgen index	6.43 ± 4.09	1.75 ± 0.96	< 0.01	< 0.01

Values are presented as mean ± standard deviation or n (%).

*PCOS* polycystic ovary syndrome, *NAFLD* non-alcoholic fatty liver disease, *LFS* liver fat score, *FLI* fatty liver index, *HSI* hepatic steatosis index, *BMI* body mass index, *WC* waist circumference, *SBP* systolic blood pressure, *DBP* diastolic blood pressure, *AST* Aspartate transaminase, *ALT* alanine aminotransferase, *GGT* Gamma-glutamyl transferase, *TG* Triglycerides, *HOMA-IR* Homeostatic model assessment for insulin resistance.

In the subjects with NAFLD assessed by LFS, 95.7% had PCOS. They were more obese and had more adverse metabolic parameters than subjects who did not have NAFLD (Table S1). They also had higher levels of total testosterone, free testosterone, and FAI. The differences in total testosterone, however, were not statistically significant after adjusting for BMI.

### PCOS, hyperandrogenism, and the risk of NAFLD

The association between NAFLD and the presence of PCOS or hyperandrogenism is presented in Table 2. In univariate analysis, the women with PCOS had 2.46 (95% CI 5.12–26.96) times higher odds for NAFLD as assessed by LFS. Other indices for NAFLD—FLI and HSI showed similar odds (OR 2.81 (95% CI 4.03–68.02) in FLI, OR 2.49 (95% CI 6.02–20.01) in HSI). After adjusting for multiple variables. including HOMA-IR, PCOS significantly increased the risk of NAFLD as assessed by FLI and HSI but not in LFS. Regarding hyperandrogenism, total testosterone level, free testosterone level, and FAI increased the odds of NAFLD as assessed by LFS, FLI, and HSI in univariate analysis. After adjusting for multiple variables, including HOMA-IR, free testosterone level and FAI exhibited statistical significance but not in total testosterone levels assessed by LFS and FLI.

**Table 2:** Logistic regression analysis of the risk of NAFLD.

	Crude OR (95% CI)	*P* value	Adjusted OR (95% CI)	*P* value
LFS				
Presence of PCOS (no = reference)	2.46 (5.12–26.96)	< 0.01	1.10 (0.80–11.35)	0.10
Total testosterone (ng/dL)	0.02 (1.01–1.03)	< 0.01	0.01 (0.99–1.02)	0.69
Free testosterone (ng/dL)	2.15 (5.74–12.76)	< 0.01	1.21 (1.70–6.56)	< 0.01
FAI	0.27 (1.24–41.37)	< 0.01	0.13 (1.05–1.22)	< 0.01
FLI				
Presence of PCOS (no = reference)	2.81 (4.03–68.02)	< 0.01	2.37 (1.41–81.61)	0.02
Total testosterone (ng/dL)	0.02 (1.01–1.03)	< 0.01	0.01 (0.99–1.02)	0.73
Free testosterone (ng/dL)	1.81 (3.96–9.50)	< 0.01	1.20 (1.86–5.87)	< 0.01
FAI	0.20 (1.16–1.28)	< 0.01	0.14 (1.08–1.22)	< 0.01
HIS				
Presence of PCOS (no = reference)	2.49 (6.02–20.01)	< 0.01	1.74 (3.01–10.76)	< 0.01
Total testosterone (ng/dL)	0.02 (1.01–1.03)	< 0.01	0.02 (1.01–1.02)	< 0.01
Free testosterone (ng/dL)	2.03 (5.31–10.88)	< 0.01	1.37 (2.62–5.91)	< 0.01
FAI	0.25 (1.23–1.35)	< 0.01	0.16 (1.12–1.24)	< 0.01

Each row represents a separate regression analysis.

Adjusting variables.

LFS: age, BMI, and HOMA-IR.

FLI: age, fasting glucose, AST, ALT, and HOMA-IR.

HSI: age, fasting glucose, HOMA-IR, TG.

*NAFLD* Non-alcoholic fatty liver disease, *OR* odds ratio, *CI* confidence interval, *LFS* Liver fat score, *PCOS* Polycystic ovary syndrome, *FAI* free androgen index, *FLI* Fatty liver index, *HSI* Hepatic steatosis index, *BMI* body mass index, *HOMA-IR* Homeostatic model assessment for insulin resistance, *AST* Aspartate transaminase, *ALT* alanine aminotransferase, *TG* Triglycerides.

### Hyperandrogenism and the risk of NAFLD in PCOS

In women with PCOS, both free testosterone level and FAI significantly increased the risk of all three NAFLD indices (all *P* < 0.01, Table 3). After adjusting for multiple variables, including HOMA-IR, statistical significance remained in all three NAFLD indices—LFS (OR 3.18 (95% CI 1.53–6.63) in free testosterone; OR 1.12 (95% CI 1.04–1.22) in FAI), FLI (OR 2.68 (95% CI 1.43–5.03) in free testosterone; OR 1.13 (95% CI 1.06–1.20) in FAI), and HSI (OR 3.29 (95% CI 2.08–5.21) in free testosterone, OR 1.15 (95% CI 1.09–1.21) in FAI). In contrast, total testosterone was not associated with any NAFLD index.

**Table 3:** Logistic regression analysis of the risk of NAFLD in women with PCOS.

	Crude OR (95% CI)	*P* value	Adjusted OR (95% CI)	*P* value
LFS				
Total testosterone (ng/dL)	1.00 (0.99–1.01)	0.55	1.00 (0.98–1.02)	0.65
Free testosterone (ng/dL)	6.55 (4.16–10.31)	< 0.01	3.18 (1.53–6.63)	< 0.01
FAI	1.25 (1.19–1.32)	< 0.01	1.12 (1.04–1.22)	< 0.01
FLI				
Total testosterone (ng/dL)	1.00 (0.98–1.01)	0.82	0.99 (0.97–1.01)	0.27
Free testosterone (ng/dL)	4.66 (2.86–7.59)	< 0.01	2.68 (1.43–5.03)	< 0.01
FAI	1.18 (1.13–1.24)	< 0.01	1.13 (1.06–1.20)	< 0.01
HSI				
Total testosterone (ng/dL)	1.00 (0.99–1.01)	0.47	1.00 (0.99–1.01)	0.54
Free testosterone (ng/dL)	4.82 (3.18–7.32)	< 0.01	3.29 (2.08–5.21)	< 0.01
FAI	1.21 (1.15–1.27)	< 0.01	1.15 (1.09–1.21)	< 0.01

Each row represents a separate regression analysis.

Adjusting variables.

LFS: age, BMI, and HOMA-IR.

FLI: age, fasting glucose, AST, ALT, and HOMA-IR.

HSI: age, fasting glucose, HOMA-IR, TG.

*NAFLD* Non-alcoholic fatty liver disease, *PCOS* Polycystic ovary syndrome, *OR* odds ratio, *CI* confidence interval, *LFS* Liver fat score *FAI* free androgen index, *FLI* Fatty liver index, *HSI* Hepatic steatosis index, *BMI* body mass index, *HOMA-IR* Homeostatic model assessment for insulin resistance, *AST* Aspartate transaminase, *ALT* alanine aminotransferase, *TG* Triglycerides.

We conducted a subgroup analysis stratified by BMI and insulin resistance (Table 4). Among overweight or obese subjects, free testosterone level and FAI significantly increased the odds of NAFLD as assessed by LFS (OR 4.12 (95% CI 1.72–9.88) in free testosterone, OR 1.14 (95% CI 1.04–1.23) in FAI) after adjusting for variables. When subjects were stratified by insulin resistance, subjects who had higher insulin resistance showed a statistically significant association between free testosterone level and FAI and NAFLD assessed by LFS (OR 3.46 (95% CI 1.49–8.05) in free testosterone, OR 1.12 (95% CI 1.03–1.22) in FAI) after adjusting variables. When applying other NAFLD indices, FLI and HSI showed similar trends, except in the case of non-insulin resistant subjects assessed by HSI—both free testosterone and FAI were significantly associated with NAFLD (Tables S2 and S3).

**Table 4:** Subgroup analysis for the risk of NAFLD assessed by LFS in women with PCOS.

	Crude OR (95% CI)	*P* value	Adjusted OR (95% CI)	*P* value
A. BMI				
Nonobese (BMI < 23 kg/m^2^) (n = 367, n of NAFLD = 11)				
Total testosterone (ng/dL)	0.97 (0.93–1.02)	0.21	0.95 (0.90–1.01)	0.12
Free testosterone (ng/dL)	0.01 (1.01–11.20)	0.05	1.49 (0.33–6.82)	0.61
FAI	1.08 (0.86–1.35)	0.52	0.95 (0.70–1.28)	0.72
Overweight or obesity (BMI ≥ 23 kg/m^2^) (n = 300, n of NAFLD = 142)				
Total testosterone (ng/dL)	1.01 (0.99–1.02)	0.32	1.00 (0.98–1.02)	0.87
Free testosterone (ng/dL)	3.44 (1.98–5.95)	< 0.01	4.12 (1.72–9.88)	< 0.01
FAI	1.15 (1.09–1.22)	< 0.01	1.14 (1.04–1.23)	< 0.01

	Crude OR	*P* value	Adjusted OR	*P* value
B. HOMA-IR				
HOMA-IR < 2.00 (n = 378, n of NAFLD = 7)				
Total testosterone (ng/dL)	0.99 (0.94–1.04)	0.62	0.99 (0.95–1.05)	0.99
Free testosterone (ng/dL)	2.99 (0.37–13.3)	0.15	3.27 (0.55–19.62)	0.20
FAI	1.16 (0.97–1.39)	0.11	1.16 (0.92–1.47)	0.22
HOMA-IR ≥ 2.00 (n = 289, n of NAFLD 126)				
Total testosterone (ng/dL)	1.00 (0.99–1.01)	0.95	1.00 (0.97–1.02)	0.69
Free testosterone (ng/dL)	1.75 (3.14–10.57)	< 0.01	3.46 (1.49–8.05)	< 0.01
FAI	1.21 (1.13–1.30)	< 0.01	1.12 (1.03–1.22)	0.01

Adjusting for age, BMI and HOMA-IR.

*NAFLD* Non-alcoholic fatty liver disease, *LFS* Liver fat score, *PCOS* Polycystic ovary syndrome, *OR* odds ratio, *CI* confidence interval, *BMI* body mass index, *FAI* free androgen index, *HOMA-IR* Homeostatic model assessment for insulin resistance.

## Discussion

In this study, we found that women with PCOS exhibited a higher prevalence of NAFLD as assessed by LFS, FLI, and HSI than controls, and it was independent of age, BMI, and insulin resistance. In women with PCOS, free testosterone level and FAI were associated with NAFLD, but total testosterone level was not associated with any NAFLD index. This association was more prominent in obese women and women who had higher insulin resistance.

The prevalence of NAFLD in women with PCOS was higher than in the control group, but varied widely depending on the specific index of NAFLD (19.9% vs. 2.1% by LFS, 10.3% vs. 0.7% by FLI, and 32.3% vs. 4.2% by HSI). Among several studies conducted in Asian populations, a Chinese study reported that the prevalence of NAFLD assessed by ultrasonography was 56.2% in women with PCOS, and 38.0% in controls^24^. A Korean study, including only non-obese women, reported that the prevalence of NAFLD as assessed by ultrasonography was 5.5% in women with PCOS and 2.8% in control women^25^. Our study population was younger and non-obese, and had lower HOMA-IR than the Chinese study population, and included both obese and non-obese women compared to the Korean study. Therefore, the prevalence of NAFLD in our study population, that is, 10–30%, is reasonable. Although the validation of each NAFLD index for an Asian population had previously been performed^26,27^, the PCOS population was not included. There is one study assessing NAFLD in women with PCOS by HSI from Italy^10^. The study reported that the prevalence of NAFLD was 68.8% in women with PCOS and 33.3% in controls^10^. Compared to our study population, the subjects in the Italian study had a different ethnicity and were older, more obese, and metabolically unhealthy. Lind et al*.* compared the predictability of NAFLD indices to that of magnetic resonance imaging-proton density fat fraction in Caucasians and found that FLI was suitable in a population-based sample and LFS was best in high-risk populations^20^. Further study is warranted to validate the capacity of NAFLD indices to predict NAFLD in the PCOS population.

We found an independent association between hyperandrogenism by free testosterone and FAI and NAFLD assessed by all three indices in the total study population as well as in the PCOS population. The total testosterone level, however, was not associated with any index of NAFLD. This finding is consistent with a previous study, which reported that, in 2117 community-dwelling women, free testosterone was associated with NAFLD, while total testosterone was not^28^. Free testosterone is calculated from the total testosterone level and SHBG. SHBG regulates hepatic lipogenesis by reducing acetyl-CoA-carboxylase levels^29^. In NAFLD subjects, SHBG mRNA expression and circulating SHBG levels were shown to be decreased^30^. One meta-analysis demonstrated that in women, the total testosterone level was not associated with NAFLD, while low SHBG was associated with an increased risk of NAFLD^31^. Taken together, the results suggest that while screening of PCOS patients for NAFLD is performed, free testoster one level or FAI, but not total testosterone level, should be evaluated.

Insulin resistance plays a crucial role in the development and progression of both NAFLD and PCOS. Hyperinsulinemia by insulin resistance increases hepatic de novo lipogenesis by increasing diacylglycerol content and induces inflammation and fibrosis by producing reactive oxygen species in the liver^32,33^. In PCOS, insulin resistance makes ovarian theca cells sensitized to luteinizing hormone and produce more androstenedione^34^. Nevertheless, in our study, after adjusting for insulin resistance, we found an independent association between hyperandrogenism and NAFLD in PCOS. It is controversial whether hyperandrogenism itself affects NAFLD or perpetuates insulin resistance, leading to the development of NAFLD. Jones et al*.* reported that women with PCOS and hyperandrogenemia (FAI > 7.0%) had higher liver fat content compared to women with PCOS and normoandrogenemia after adjusting for HOMA-IR^7^. Even though an accurate diagnostic tool, MRI, was used for NAFLD diagnosis, the size of the study population was small, with 19 hyperandrogenic PCOS subjects and 10 normoandrogenic PCOS subjects. Furthermore, when evaluating the association between hyperandrogenism and liver fat content in MRI, the data were not adjusted for BMI and only adjusted for HOMA-IR. Kumarendran et al. utilized a primary care database in the United Kingdom and found that hyperandrogenemia (total testosterone > 3.0 nmol/L) independently increased the risk of NAFLD in PCOS^8^. The data were adjusted for BMI, diabetes, or impaired glucose regulation, but not for insulin resistance. In the current study, we adjusted for multiple factors that could affect NAFLD, including insulin resistance, and found an independent association between hyperandrogenism and NAFLD in PCOS. In contrast, there are studies reporting that the relationship between hyperandrogenism and NAFLD in PCOS patients is not independent but mediated by insulin resistance. Macut et al. conducted a study with 600 Caucasian women with PCOS and performed a multivariate logistic regression analysis, revealing that NAFLD defined by LFS was independently associated with HOMA-IR, but not with FAI^9^. Petta et al. recruited 202 Italian PCOS women and 101 age-matched controls, and found that NAFLD assessed by HSI was independently associated with insulin sensitivity index while FAI showed no statistical significance except in nonobese group^10^. Since current cross-sectional studies are insufficient to establish a causal relationship, further prospective studies are warranted to elucidate the causal impact of hyperandrogenism, independent of insulin resistance, on the development of NAFLD in PCOS.

Interestingly, subgroup analysis showed that the association between hyperandrogenism and NAFLD was prominent in the overweight and/or obesity group and the more insulin-resistant group. It could be speculated that a large amount of fat and insulin resistance could augment the role of hyperandrogenemia in developing NAFLD. However, it should be noted that the number of NAFLD cases in nonobese and lower HOMA-IR groups was small so that clinical significance might not be observed. Additionally, the overweight and/or obese group also featured a significant association between free testosterone and FAI and NAFLD. A previous study from Italy found that FAI was associated with NAFLD in obese women with PCOS but not in nonobese women with PCOS after adjusting for multiple variables, including the insulin sensitivity index^10^. Recognizing those high-risk population and implementing early intervention strategies in PCOS, along with investigating into the biochemical mechanisms, are needed to validate these findings.

The strength of our study is that various NAFLD indices and testosterone markers were comprehensively assessed in young women with PCOS. In addition, the association between hyperandrogenism and NAFLD, independent of insulin resistance, was probed by adjusting for HOMA-IR. Our study also carries several limitations. First, even though the three NAFLD indices that we used in the study have been validated in many studies, they are not a gold standard for the diagnosis of NAFLD. Additionally, we were unable to utilize other NAFLD indices such as the NAFLD fibrosis core, FIB-4, or Framingham steatosis index due to the lack of required variables or low reproducibility in Korean population. Second, we utilized HOMA-IR as an indicator for insulin resistance but did not perform an insulin clamp study. Third, our study is a cross-sectional study, and we could not, therefore, establish a causal relationship between hyperandrogenism and NAFLD. Fourth, as our study was performed in you Asian women, there might be a potential selection bias. Further research is needed to generalize the findings to a large age range or different ethnicities. Last, we measured testosterone levels using a chemiluminescent immunoassay, which is generally recognized to have lower accuracy compared to methods utilizing mass spectrometry. Nevertheless, the importance of our study is the establishment of free testosterone level and FAI as important markers for early detection of NAFLD in young women with PCOS.

In conclusion, PCOS was related to NAFLD as measured by the NAFLD indices, LFS, FLI, and HSI. Free testosterone levels and FAI were independently associated with NAFLD in PCOS, but total testosterone levels were not. Our results indicate that, in young women with PCOS, the risk of NAFLD is higher than controls of the same age, and both free testosterone levels and FAI might be important predictors for NAFLD even after adjusting for multiple risk factors, including insulin resistance. Also, it is important to note that in the clinical management of PCOS patients, particularly those who are obese or have high insulin resistance, there appears to be a stronger association between hyperandrogenism and NAFLD. Further studies are needed to validate the association, elucidate the underlying mechanism mediating hyperandrogenism and NAFLD, and establish intervention and prevention strategies for these conditions.

### Supplementary Information

Supplementary Tables.

## Data Availability

The datasets analyzed during the current study are available from the corresponding author on reasonable request.

## Abbreviations

ALT: Alanine aminotransferase
AST: Aspartate transaminase
BMI: Body mass index
FAI: Free androgen index
FLI: Fatty liver index
GGT: Gamma-glutamyl transferase
HOMA-IR: Homeostatic model assessment for insulin resistance
HSI: Hepatic steatosis index
LFS: Liver fat score
NAFLD: Non-alcoholic fatty liver disease
NICHD: National Institute of Child Health and Human Disease
PCOS: Polycystic ovary syndrome
SHBG: Sex hormone-binding globulin
TG: Triglycerides
