# Supplementary material for: Non-alcoholic fatty liver disease is associated with hyperandrogenism in women with polycystic ovary syndrome
Source: Sci Rep. 2023 Aug 17;13:13397. doi: 10.1038/s41598-023-39428-4 (PMC10435477; doi:10.1038/s41598-023-39428-4)
Supplement: Supplementary file 1 — Supplementary Tables. [file 41598_2023_39428_MOESM1_ESM.docx]

**Supplementary Table 1.** Basal characteristics of study subjects according to NAFLD assessed by liver fat score

|  | NAFLD  (n = 139) | Non-NAFLD  (n = 817) | *P* value  (unadjusted) | *P* value  (BMI-adjusted) |
| --- | --- | --- | --- | --- |
| PCOS (n (%)) | 133 (95.7) | 534 (65.4) | <0.01 | <0.01 |
| NAFLD-related index |  |  |  |  |
| LFS | 1.46 ± 2.20 | −2.26 ± 0.75 | <0.01 | <0.01 |
| FLI | 52.6 ± 27.8 | 8.61 ± 12.47 | <0.01 | <0.01 |
| HSI | 41.0 ± 6.5 | 30.8 ± 4.4 | <0.01 | <0.01 |
| Age (years) | 26 ± 6 | 25 ± 5 | <0.01 | 0.03 |
| BMI (kg/m^2^) | 29.1 ± 4.6 | 21.7 ± 3.2 | <0.01 | <0.01 |
| WC (cm) | 92.1 ± 10.6 | 74 ± 8.2 | <0.01 | <0.01 |
| SBP (mmHg) | 122 ± 13 | 109 ± 10 | <0.01 | <0.01 |
| DBP (mmHg) | 85 ± 8 | 69 ± 8 | <0.01 | <0.01 |
| AST (U/L) | 38 ± 29 | 20 ± 5 | <0.01 | 0.01 |
| ALT (U/L) | 46 ± 46 | 17 ± 7 | <0.01 | <0.01 |
| GGT (U/L) | 28 ± 18 | 13 ± 6 | <0.01 | <0.01 |
| TG (mg/dL) | 150 ± 76 | 81 ± 40 | <0.01 | <0.01 |
| Fasting glucose (mg/dL) | 96 ± 24 | 85 ± 8 | <0.01 | <0.01 |
| Fasting insulin (uU/mL) | 20.5 ± 11.0 | 6.9 ± 3.8 | <0.01 | <0.01 |
| HOMA-IR | 4.77 ± 2.98 | 1.46 ± 0.82 | <0.01 | <0.01 |
| Total testosterone(ng/dL) | 75.24 ± 22.34 | 65.53 ± 22.68 | <0.01 | 0.06 |
| Free testosterone (ng/dL) | 1.39 ± 0.56 | 0.81 ± 0.44 | <0.01 | <0.01 |
| Free androgen index | 9.47 ± 5.66 | 4.26 ± 3.18 | <0.01 | <0.01 |

**Supplementary Table 2.** Subgroup analysis for the risk of NAFLD assessed by FLI in women with PCOS

A. HOMA-IR

|  | Crude OR | *P* value | Adjusted OR | *P* value |
| --- | --- | --- | --- | --- |
| HOMA-IR < 2.00 (n = 378, n of NAFLD = 8) | | | | |
| Total testosterone (ng/dL) | 0.95 (0.90–1.00) | 0.06 | 0.98 (0.92–1.05) | 0.55 |
| Free testosterone  (ng/dL) | 3.18 (0.80–12.68) | 0.10 | 3.49 (0.60–20.18) | 0.16 |
| FAI | 1.23 (1.05–1.44) | 0.01 | 1.38 (1.07–1.77) | 0.02 |
| HOMA-IR ≥ 2.00 (n = 289, n of NAFLD 126) | | | | |
| Total testosterone (ng/dL) | 1.01 (0.99–1.02) | 0.43 | 1.00 (0.98–1.02) | 0.86 |
| Free testosterone  (ng/dL) | 3.29 (1.88–5.76) | <0.01 | 2.58 (1.28–5.16) | 0.01 |
| FAI | 1.12 (1.06–1.18) | <0.01 | 1.10 (1.03–1.18) | <0.01 |

Adjusting for age, fasting glucose, AST, ALT, and HOMA-IR.

**Supplementary Table 3.** Subgroup analysis for the risk of NAFLD assessed by HSI (hepatic steatosis index) in women with PCOS

A. BMI

|  | Crude OR | *P* value | Adjusted OR | *P* value |
| --- | --- | --- | --- | --- |
| **Nonobese (BMI < 23 kg/m^2^) (n = 367, n of NAFLD = 31)** | | | | |
| Total testosterone (ng/dL) | 1.00 (0.97–1.02) | 0.77 | 1.00 (0.97–1.02) | 0.75 |
| Free testosterone  (ng/dL) | 1.00 (0.34–2.95) | 0.99 | 0.80 (0.27–2.35) | 0.69 |
| FAI | 0.90 (0.73–1.09) | 0.25 | 0.86 (0.69–1.06) | 0.15 |
| **Overweight or obesity (BMI ≥ 23 kg/m^2^) (n = 300, n of NAFLD = 184)** | | | | |
| Total testosterone (ng/dL) | 1.01 (0.99–1.02) | 0.13 | 1.01 (0.99–1.02) | 0.41 |
| Free testosterone  (ng/dL) | 2.72 (1.55–4.79) | <0.01 | 2.10 (1.13–3.90) | 0.02 |
| FAI | 1.14 (1.07–1.21) | <0.01 | 1.07 (1.01–1.13) | 0.04 |

B. HOMA-IR

|  | Crude OR | *P* value | Adjusted OR | *P* value |
| --- | --- | --- | --- | --- |
| **HOMA-IR < 2.00 (n = 378, n of NAFLD = 61)** | | | | |
| Total testosterone (ng/dL) | 1.00 (0.99–1.02) | 0.94 | 1.01 (0.99–1.03) | 0.33 |
| Free testosterone  (ng/dL) | 2.58 (1.45–4.59) | <0.01 | 2.85 (1.32–6.15) | 0.01 |
| FAI | 1.10 (1.03–1.16) | <0.01 | 1.12 (1.01–1.25) | 0.03 |
| **HOMA-IR ≥ 2.00 (n = 289, n of NAFLD 154)** | | | | |
| Total testosterone (ng/dL) | 1.00 (0.99–1.01) | 0.71 | 1.00 (0.98–1.01) | 0.46 |
| Free testosterone  (ng/dL) | 3.94 (2.19–7.08) | <0.01 | 3.33 (1.79–6.18) | <0.01 |
| FAI | 1.17 (1.10–1.25) | <0.01 | 1.15 (1.07–1.23) | <0.01 |

Adjusting for age, fasting glucose, HOMA-IR, TG.
